# Development and Optimization of Nanolipid-Based Formulation of Diclofenac Sodium: In Vitro Characterization and Preclinical Evaluation

**DOI:** 10.3390/pharmaceutics14030507

**Published:** 2022-02-25

**Authors:** Ameeduzzafar Zafar, Nabil K Alruwaili, Syed Sarim Imam, Mohd Yasir, Omar Awad Alsaidan, Ali Alquraini, Alenazy Rawaf, Bader Alsuwayt, Md. Khalid Anwer, Sultan Alshehri, Mohammed M. Ghoneim

**Affiliations:** 1 Department of Pharmaceutics, College of Pharmacy, Jouf University, Sakaka 72341, Al-Jouf, Saudi Arabia; nkalruwaili@ju.edu.sa (N.K.A.); osaidan@ju.edu.sa (O.A.A.); 2 Department of Pharmaceutics, College of Pharmacy, King Saud University, Riyadh 11451, Saudi Arabia; salshehri1@ksu.edu.sa; 3 Department of Pharmacy, College of Health Science, Arsi University, Asella 396, Ethiopia; mohdyasir31@gmail.com; 4 Department of Pharmaceutical Chemistry, Faculty of Clinical Pharmacy, Al Baha University, Al Baha 65779, Saudi Arabia; aalquraini@bu.edu.sa; 5 Department of Medical Laboratory, College of Applied Medical Sciences-Shaqra, Shaqra University, Shaqra 11961, Saudi Arabia; ralenazy@su.edu.sa; 6 Department of Pharmacy Practice, College of Pharmacy, University of Hafr Al-Batin, Hafr Al-Batin 31991, Saudi Arabia; balsuwayt@uhb.edu.sa; 7 Department of Pharmaceutics, College of Pharmacy, Prince Sattam Bin Abdulaziz University, Al-kharj 11942, Saudi Arabia; m.anwer@psau.edu.sa; 8 Department of Pharmacy Practice, College of Pharmacy, Almaarefa University, Ad Diriyah 13713, Saudi Arabia; mghoneim@mcst.edu.sa

**Keywords:** bilosomes, diclofenac, optimization, pharmacokinetic, pharmacodynamic study

## Abstract

In the present research study, we formulate bilosomes (BMs) of diclofenac (DC) for oral delivery for enhancement of therapeutic efficacy (anti-inflammatory disease). The BMS were prepared by thin film hydration method and optimized by Box–Behnken design (BBD) using cholesterol (A), lipid (B), surfactant (C), and bile salt (D) as formulation factors. Their effects were evaluated on vesicle size (Y_1_) and entrapment efficacy (Y_2_). The optimized DC-BMs-opt showed a vesicle size of 270.21 ± 3.76 nm, PDI of 0.265 ± 0.03, and entrapment efficiency of 79.01 ± 2.54%. DSC study result revealed that DC-BMs-opt exhibited complete entrapment of DC in BM matrix. It also depicted significant enhancement (*p* < 0.05) in release (91.82 ± 4.65%) as compared to pure DC (36.32 ± 4.23%) and DC-liposomes (74.54 ± 4.76%). A higher apparent permeability coefficient (2.08 × 10^−3^ cm/s) was also achieved compared to pure DC (6.6 × 10^−4^ cm/s) and DC-liposomes (1.33 × 10^−3^ cm/s). A 5.21-fold and 1.43-fold enhancement in relative bioavailability was found relative to pure DC and DC liposomes (DC-LP). The anti-inflammatory activity result showed a significant (*p* < 0.05) reduction of paw edema swelling compared to pure DC and DC-LP. Our findings revealed that encapsulation of DC in BMs matrix is a good alternative for improvement of therapeutic efficacy.

## 1. Introduction

Diclofenac (DC) is a non-steroidal anti-inflammatory (NSAID) agent with anti-inflammatory and antipyretic activity. It is widely used for treatment of acute pain and various anti-inflammatory diseases such as osteoarthritis and rheumatoid arthritis. It has a short half-life of 1–2 h due to extensive first-pass metabolism. It belongs to the BCS-II class drug and reported low solubility [1]. The long exposure to DC inhibits prostaglandin formation which causes gastric irritation, bleeding, and ulcers [2]. These side effects can be minimized by reducing direct contact of drug with GIT [3].

Various studies have been published to overcome complications of gastric irritation and ulcers. Various types of formulation have been reported to increase therapeutic efficacy and side effects of DC. A diclofenac-loaded nanoformulation was prepared and evaluated for different parameters [4]. They reported a nanometric size with high entrapment efficiency. A significant effect was observed on pharmacokinetic and pharmacodynamic activities. The low dose depicted clinical therapeutic levels in blood for up to 120 h, with minimal drug accumulation in organs as well as better efficacy than other controls. Akbari et al. developed transdermal diclofenac niosomal gel for improvement of therapeutic activity. The prepared formulation showed nano-metric size, negative zeta-potential, and high entrapment efficiency. The biological activity result revealed significantly lower licking time than conventional formulation [5]. In another study, diclofenac sodium-loaded nanovesicles were prepared by double solvent displacement method [6]. The prepared liposomes showed nano-metric size, negative zeta potential, and high encapsulation efficiency. The permeation results revealed a higher transdermal passage of drug. Gaur et al. prepared diclofenac sodium-loaded lipid vesicles and analyzed them for physical and biological activity [7]. The prepared formulation depicted more than 90% release with an enhanced pharmacokinetic profile. 

The application of lipid-based nanoformulations is rising as an effective method for drug delivery. It can enhance drug solubility as well as bioavailability and reduce side effects. There are various lipid nanoformulations such as solid lipid nanoparticles [8,9], nanostructured lipid carriers [10], liposomes [11], and bilosomes [12]. Among them, bilosomes (BMs) are the new nano-sized lipid vesicular formulation used for different therapeutic agents. They are an elastic vesicular system composed of phospholipid, surfactant, cholesterol, and bile salt [13]. The bile salt enters into the lipid bilayer and lowers the phase transition temperature and builds vesicles deformable under body temperature [14]. They have been reported to enhance bioavailability of many drugs [15,16]. They show less drug leakage and high lymphatic drug transport as compared to niosomes and liposomes. They also prevent the enzymatic degradation in gastrointestinal tract and pass from reticular endothelial system [17]. In the GIT, bile salt that breaks vesicle before reaching to target site [18]. The bile salt acts as a solubilizing and permeation enhancer agent and may improve bioavailability of poorly soluble drugs [19]. Different types of bile salts such as sodium deoxycholate, sodium glycolate, sodium taurocholate, and sodium glycolate are used to prepare BMs. Among them, sodium deoxycholate is commonly used for formulation of BMs due to its nontoxic nature [20]. Shukla et al. formulated a diphtheria toxoid-loaded BM for oral administration. It produced quantifiable anti-diphtheria toxoid response in serum as well as mucosal secretion [21]. In another study, Shukla et al. formulated BM oral delivery of hepatitis B and produced concentration level of systemic and mucosal antibodies [22]. Zakaria et al. formulated piperine-loaded BMs for antiviral and anti-inflammatory activity [23]. Piperine-loaded BMs exhibited significantly reduced oxidant markers and cytokines in MERS-Co-V infected mice compared to pure piperine. El Taweel et al. formulated zolmitriptan-loaded BMs in situ gel for nose brain delivery [24]. BMs in situ gel produced significant bioavailability (1176.9%) compared to BMs dispersion (835.7%).

Up until now, no study has been performed to evaluate efficacy of diclofenac bilosomes (DC-BMs) to improve therapeutic efficacy. The objective of study is to prepare and optimize them using experimental design software (Stat-Ease, Minneapolis, MN, USA). The selected formulation (DC-BMs-opt) was evaluated for physicochemical characterization, in vitro, ex vivo study, and pharmacokinetic and pharmacodynamic study.

## 2. Experimental

### 2.1. Materials

Diclofenac potassium, lipid (L-α-Phosphatidylcholine), pluronic F123, cholesterol, and bile salt (sodium deoxycholate) were procured from Sigma Aldrich (St. Louis, MO, USA). Dialysis bag (MWCO 12,000 kDa) was procured from HiMedia laboratory (Mumbai, India). HPLC grade water, acetonitrile, and methanol were obtained from SD-fine chemicals (Mumbai, India). 

### 2.2. Methods

#### 2.2.1. Formulation of Bilosomes

DC-BMs were prepared by slightly modified thin-film hydration method [13]. The lipid, surfactant, and cholesterol with a fixed dose of DC were taken in different ratios and dissolved in organic solvent (methanol: chloroform) as shown in Table 1. The solution was transferred to a round bottom flask and then organic solvent was evaporated at a temperature of 50 °C with reduced pressure using a rotary evaporator (IKA, RV-3V, Staufen, Germany). A thin lipid film was formed on wall of flask and stored in a desiccator for 24 h to remove moisture. The film was hydrated with phosphate buffer (10 mL) containing sodium deoxycholate for 3 h. The dispersion was collected and sonicated for 15 min to reduce size. The prepared formulations were collected and stored at 4 °C for further study.

#### 2.2.2. Optimization

DC-BMs were optimized by using 4 factors at 3 level Box–Behnken design (BBD). The independent variables cholesterol (A), lipid (B), pluronic F127 (C), and bile salt (D) were taken as independent factors and their effects were assessed on VS (R_1_) and EE (R_2_). The design showed twenty-seven formulations with five center points from software. The practical value of dependent variables (VS as R_1_ and EE as R_2_). The data were fitted into software and evaluated for different models, i.e., linear, 2nd order, and quadratic models to determine best fit model. The regression analysis and ANOVA of best fit model were applied. The three-dimensional plots (3D plots) were plotted to interpret the effect of each factor over each response. 

#### 2.2.3. Bilosomes Evaluation

The prepared DC-BMs (F1-F27) vesicle size (VS), PDI, and zeta potential (ZP) were measured by size analyzer (Zeta sizer Nano S90, Malvern, UK) at 25 °C. The diluted DC-BMs were placed in quartz cuvette and their size and PDI were measured. The same samples were evaluated for Zeta potential by using cuvette with an electrode. 

#### 2.2.4. Entrapment Efficiency (EE)

EE of DC in prepared BMs was analyzed by indirect method [25]. The prepared DC-BMs (2 mL) were taken in a tube and samples centrifuged at 6000 rpm. The supernatants were collected and diluted and DC content was measured using a UV spectrophotometer. EE in each sample was calculated by using formula:

(1) $$\mathrm{EE}\;=\;\frac{\mathrm{Total}\;\mathrm{DC}\;-\;\mathrm{DC}\;\mathrm{in}\;\mathrm{supernatant}}{\mathrm{Total}\;\mathrm{DC}\;}\times \;100$$

#### 2.2.5. Surface Morphology

The surface morphology of an optimized bilosomes (DC-BMs-opt) was examined by transmission electron microscopy (TEM, Philips CM 10, Eindhoven, The Netherlands). One drop of diluted sample was placed over grid and stained with phosphotungistic acid. The grid was air-dried and placed into instrument, and image was captured.

#### 2.2.6. Thermal Analysis

DSC analysis of DC, lipid, cholesterol (CHO), Pluronic F127, SC, physical mixture and optimized formulation DC-BMs-opt was analyzed using DSC instrument (Mettler Toledo, South Miami, FL, USA). Each sample (5 mg) was taken, packed into an aluminum pan, and scanned between 25–400 °C under an inert condition. The thermograms were recorded and compared to each other. 

### 2.3. In Vitro Drug Release

The release study was performed using a pretreated dialysis bag. The test samples of pure DC, DC-LP and DC-BMs-opt (equivalent to 3 mg DC) were filled into a dialysis bag and tied from both ends. The bag was immersed into release media (500 mL, phosphate buffer pH 6.8) and assembly fixed at a temperature of 37 ± 0.5 °C with stirring speed of 50 rpm. 5 mL of released content was withdrawn at a fixed time and filled with fresh release media to maintain the volume. The absorbance was measured by UV-spectrophotometer (Genesys 10S UV-Vis, Thermo-scientific, Waltham, MA, USA) at 276 nm. The release data fitted to different release kinetic models to find best fit model.

### 2.4. Ex Vivo Permeation Study

The ex vivo permeation study was done using rat intestine. The rats were kept fasted overnight (24 h), then sacrificed and intestines were collected. The intestine was washed with normal saline (0.9% NaCl) and DC-BMs-opt, DC-LP, and pure DC were filled. The intestine was then immersed into a physiological ringer solution (composition NaCl, KCl, KH_2_PO_4_, CaCl_2_, glucose) as permeation media and placed over a magnetic stirrer. The system was fixed at 37 ± 0.5 °C with a regular supply of 95% O_2_ and 5% CO_2_. At specific time, 2 mL sample was collected at a fixed time (0, 30, 60, 90, 120, and 180 min) and analyzed for drug permeation through previously developed HPLC [26]. HPLC system was run using acetonitrile and methanol (7:3) with a flow rate of 0.75 mL/min, injection volume of 20 µL, and UV-detector at 276 nm. The apparent permeability and enhancement ratio was measured.

(2) $$\mathrm{Appearent}\;\mathrm{permeability}\;=\;\frac{\mathrm{Flux}}{\mathrm{Area}\;\times \;\mathrm{Initial}\;\mathrm{drug}\;\mathrm{concentration}}$$

(3) $$\mathrm{Enhancement}\;\mathrm{ratio}\;=\;\frac{\mathrm{Permeability}\;\mathrm{coefficient}\;\mathrm{of}\;\mathrm{DC}\;\mathrm{BLopt}}{\mathrm{Permeability}\;\mathrm{coefficient}\;\mathrm{of}\;\mathrm{the}\;\mathrm{pure}\;\mathrm{DC}}$$

### 2.5. In Vivo Study

#### 2.5.1. Bioavailability Study

The study protocol was approved by institutional animal ethical committee Jouf University Sakaka, Al-Jouf, Saudi Arabia (Approval Number 04-02-43). The animals (Wistar Albino rats, 200–250 gm, either sex) were procured from an animal house. The animals were provided with free access to food and water and kept at 25 °C/50%RH. The study performed with three animal groups, each group having six rats. Group 1 was administered pure DC, Group 2 and Group 3 were administered with DC-LP and DC-BMs-opt. The samples of pure DC, DC-LP, and DC-BMs (equivalent to 2 mg/kg of DC) were administered orally to rats. At a definite time of 0, 0.5, 1, 2, 3, 6, 12, and 24 h, blood sample was collected from retro-orbital plexus into an EDTA tube. The plasma was separated by centrifuging blood sample at 5000 rpm for 15 min. The plasma was extracted by liquid-phase extraction method. The plasma was mixed with ethyl acetate and acetone (8:2, 0.5 mL), vortexed for 1 min, and then centrifuged to collect supernatant. The supernatant was dried under a stream of nitrogen and dried sample was reconstituted with acetonitrile and filtered through a 0.25 µm membrane filter. The sample (20 µL) was injected into HPLC system to calculate DC concentration in each animal.

#### 2.5.2. Pharmacodynamic Study

The rats were divided into four groups and each group containing six rats (n = 6). Group A was taken as normal control, Group B was used as disease control, Group C was administered with pure DC, group D was treated with DC-LP, and group E was treated with DC-BMs-opt. The carrageenan solution (1%, in saline) was administered to different groups by intra-plantar injection in a right hind paw to induce inflammation. The pure DC, DC-LP, and DC-BMs-opt were administered orally before 30 min of carrageenan injection. The paw volume of each rat was measured by plethysmometer (Ugo Basile, Varese, Italy), before and after carrageenan injection at different time intervals (0, 1, 2, 3, 6, 9, 12, 24 h). The degree of edema induced was assessed by following equation.

(4) $$\%\;\mathrm{Edema}\;\mathrm{inhibition}=\frac{\mathrm{Vt}-V0}{V0}\;\times \;100$$

where Vt and V0, are volume of right hind paw after and before carrageenan treatment.

### 2.6. Statistical Analysis

Data are represented as average ± SD. Graph Pad software Inc., La Jolla, CA, USA was used for statistical analysis. *p* < 0.05 was taken as statistical significance.

## 3. Result and Discussion

### 3.1. Optimization

**Figure 1:**
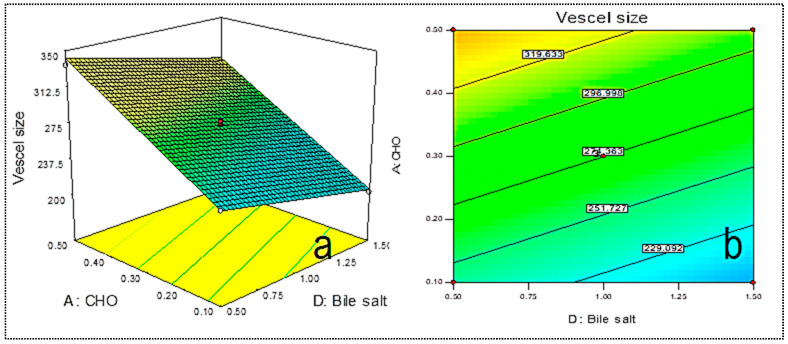
3D plot showing effect (**a**) of independent variables lipid (A), cholesterol, and bile salt (D) on vesicle size. Contour plot showing effect (**b**) of independent variables lipid (A), cholesterol, and bile salt (D) on vesicle size (R_1_).

**Figure 2:**
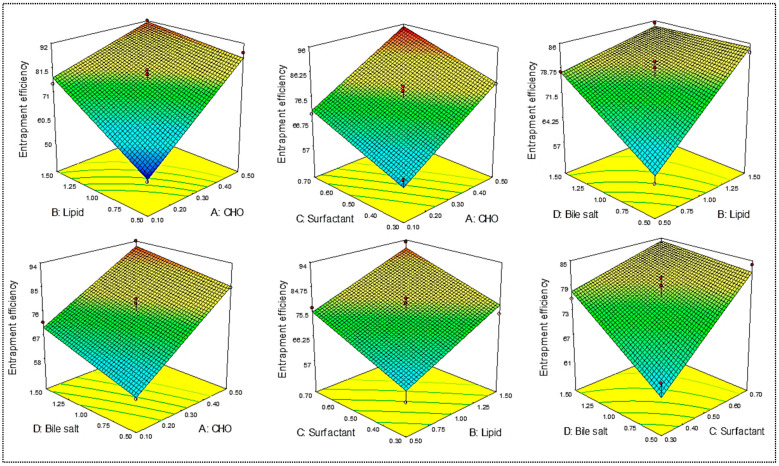
Effect of independent variables lipid (A), cholesterol (B), Pluronic F127 (C), and bile salt (D) on entrapment efficiency (R_2_).

DC-BMs were optimized by using 4-factor at 3-levels Box–Behnken design. The formulation composition of prepared DC-BMs with their dependent variables VS (R_1_) and EE (R_2_)are shown in Table 1. The minimum and maximum vesicle size was found in range of 169.34 nm (F1)–380.14 nm (F4). The lowest EE was found for formulation (F1) as 50.23% and highest found for formulation (F12) as 94.03%. The experimental value of all prepared DC-BMs was applied into experimental design model and best fit model was found linear for vesicle size and 2nd order (2F1) model for EE. The adequate precision for VS and EE found >4 and represents model as well fitted [27]. The predicted R^2^ values were found to be closer to adjusted R^2^ and statistical analysis expressed in Table 2. The ANOVA of both responses were analyzed and sum of square, mean square, F-value, and *p*-value of dependent variable is given in Table 3. 3D-plots were constructed (Figure 1 and Figure 2), and effect of an independent variable over responses were interpreted. The polynomial equation of responses was given below and it explains direct relationship of independent variables to responses. The positive and negative signs denote favorable and unfavorable effect of formulation factors over response. 

### 3.2. Effect of Independent Variables over Vesicle Size (R_1_)

The vesicle size of prepared DC-BMs was found in range of 169.34 nm (F1)–380.14 nm (F4). The formulation (F1) prepared with composition cholesterol (A, 0.1%), lipid (B, 0.5%), surfactant (C, 0.5%), and bile salt (D, 1%) showed lowest size and formulation (F4) showed maximum size with composition cholesterol (0.5%), lipid (1.5%), surfactant (0.5%), and bile salt (1%). The difference in size found due to variation in used composition. From the result, it can be observed that used independent variables have shown a significant effect. 3D response surface plot (Figure 1a,b) and polynomial Equation (5) show that increasing a CHO (A) concentration led to an increase in vesicle size due to greater amount of CHO deposited into lipid bilayer [28]. The second factor lipid concentration (B) increases vesicle size due to enhancement in viscosity of dispersion and thickness of lipid bilayers. Similar types of findings reported in reported research of papain liposomes [29], and diclofenac liposome [30]. The surfactant concentration (PP123, C) and bile salt (D) depicted a negative effect on BNs size. The enhancement in surfactant (PP123) gave the reduction in vesicle size because at high concentration of surfactant, interfacial tension reduced between lipid phases and aqueous phase. The bile salt increases flexibility of liposomes by incorporating into lipid bilayer, thereby decreasing vesicle size [31].

Vesicle size (nm, R_1_) = 274.36 + 49.20 A + 50.23 B − 30.45 C − 18.69 D (5)

The F-value fitted to linear model and value found to be 646.04 revealed that model was significantly (*p* < 0.0001) fitted. The regression coefficient of best fit model was found to be 0.9915 and it indicates lesser variation between actual and predicted value (Table 2). The ANOVA value showed that model term cholesterol (A), lipid (B), surfactant (C), and bile salt (D) were found to be significant model term (*p* < 0.0001, Table 3). The adequate precision was 85.98 (<4), revealing the close relationship between actual and experimental value [32]. The *p*-value of lack of fit is >0.05 indicated insignificant which is good for model [33].

### 3.3. Effect of Independent Variables (A, B, C, D) on Entrapment Efficiency (R_2_)

EE of DC-BMs was found between 50.23% (F1) and 93.11% (F16). The formulation (F1) prepared with composition cholesterol (A, 0.1%), lipid (B, 0.5%), surfactant (C, 0.5%), and bile salt (D, 1%) showed minimum EE. The maximum EE was shown by formulation (F19) having composition cholesterol (A, 0.5%), lipid (B, 1.5%), surfactant (C, 0.5%), and bile salt (D, 1%). There was a significant (*p* < 0.01) variation in EE was found due to variation in ratio of independent variables. The polynomial Equation (6) and 3D response surface plot (Figure 2) showed the effect of independent variables on EE. The increase in cholesterol (A) leads to enhancement in EE of DC. This effect was found due to deposition of CHO between free spaces of lipid bilayers, which reduces the flexibility, weakens the drug mobility, and reduces diffusion for DC from BMs [34,35]. The second factor lipid (B) also plays an important role on EE. The increase in lipid concentration lead to increase in EE due to enhancement in lipid viscosity. This prevents leaching of DC from lipid bilayer due to increase in hydrophobicity and longer alkyl chain length [36]. However, surfactant (C) showed a positive effect on EE of DC in BMs. The increase in surfactant concentration led to reduction in interfacial tension and increase in viscosity protects leakage of DC from BMs. The fourth variable bile salt (D, SD%) also showed a positive effect on EE. The increases in bile salt led to an increase in EE. It showed a lesser effect than surfactant. It also had surfactant-like properties—it reduced the interfacial tension and then drug easily assimilated into lipid bilayer due to enhanced solubility and flexibility [37].

EE (%, R_2_) = 76.55 + 12.17 A + 8.054 B + 6.73 C + 4.46 D − 5.25 AB − 0.40 AC − 1.25AD − 0.55 BC − 4.78BD − 3.5 CD (6)

The second order design model (2 F1) was found to be the best fit model for EE. The model F-value 36.53 implies that model was significantly fitted (*p* < 0.001). The lack of fit was found to be non-significant (*p* = 0.0942), and indicates model is well fitted. The regression coefficient of best fit model was found to be 0.958 and it indicates lesser variation between actual and predicted value (Table 2). The polynomial equation (Equation (2)) and ANOVA of best fitted model showed coded terms, i.e., A, B, C, D, AB, BD, CD, are significant (*p* < 0.05) which means these factors had a significant effect on EE of DC in BMs (Table 3). 

### 3.4. Optimized Formulation (DC-BMs-opt)

The formulation (DC-BMs-opt) was selected from point prediction of software. The composition CHO (0.3% *w*/*v*), lipid (1% *w*/*v*), surfactant (0.5% *w*/*v*), and bile salt (1% *w*/*v*) depicted an experimental vesicle size of 270.21 ± 3.76 nm and EE of 79.01 ± 2.54%. The software showed a predicted value of vesicle size of 274.36 nm and EE of 76.56%. There was non-significant variation in result observed between experimental and predicted value. The closeness in result revealed that model is valid and reproducible.

### 3.5. Vesicle Evaluation

**Figure 3:**
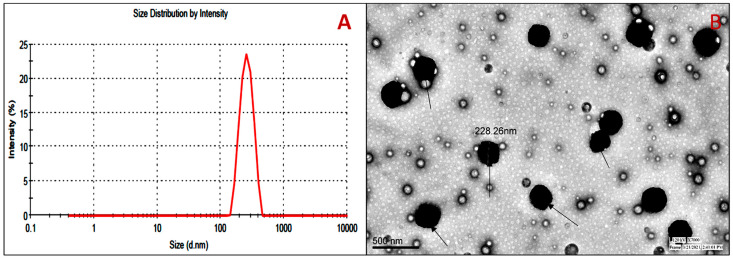
Vesicle size (**A**), and (**B**) TEM image of optimized diclofenac bilosomes (DC-BMs-opt marked with arrow).

The size of prepared DC-BMs (F1-F27) was found between 169.34 nm and 380.14 nm. The optimized composition (DC-BMs-opt) showed VS of 270.21 ± 3.76 nm (Figure 3A). PDI was found to be (0.26 ± 0.03) and revealed homogeneity of BMs-opt [38]. The zeta potential of DC-BMs-opt was of high negative (−36.34 mV) indicating that formulation was highly stable and in disaggregated form. The surface morphology exhibited spherical shape vesicles with a smooth surface without any aggregation (Figure 3B).

### 3.6. Thermal Analysis

**Figure 4:**
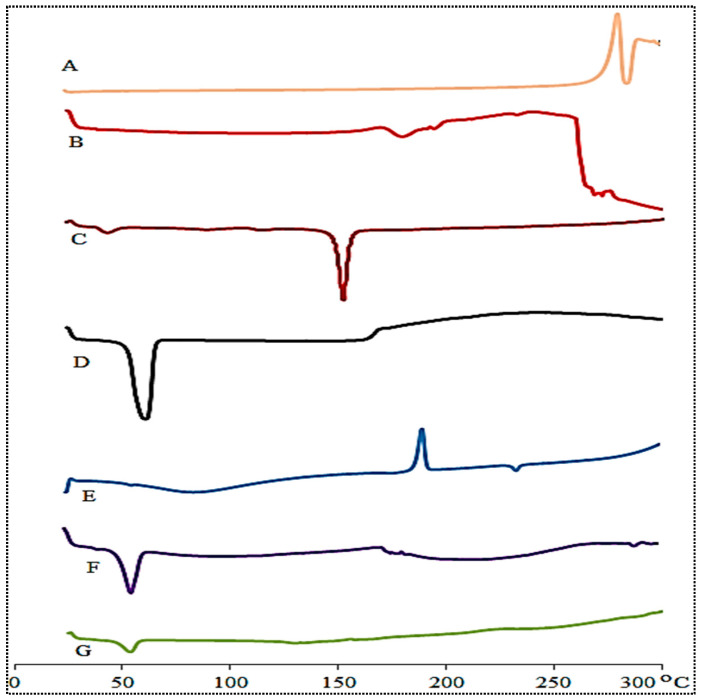
Thermal analysis of (**A**) diclofenac, (**B**) lipid, (**C**) cholesterol, (**D**) Pluronic F127, (**E**) sodium deoxycholate, (**F**), physical mixture, and (**G**) optimized diclofenac bilosomes (DC-BMs-opt).

Figure 4 shows DSC spectra of DC, lipid, CHO, Pluronic F127, SC, PM, and DC-BMs-opt. DC showed characteristic endothermic peak at 287.5 °C, which corresponds to its melting point [39]. The lipid, cholesterol, Pluronic F127, and SC exhibited a peak at 180 °C (Figure 4B), at 150 °C (Figure 4C), at 60 °C (Figure 4D), and 190 °C (Figure 4E), respectively. The physical mixture exhibited endothermic peaks at 56 °C (Pluronic F127), an exothermic peak at 180 °C, and a less intense peak of DC at 287.5 °C (Figure 4F). No characteristic endothermic peak of DC was observed in DC-BMs-opt thermogram. The observation revealed complete encapsulation or solubilization of DC into BMs matrix (Figure 4G).

### 3.7. In Vitro Drug Release

**Figure 5:**
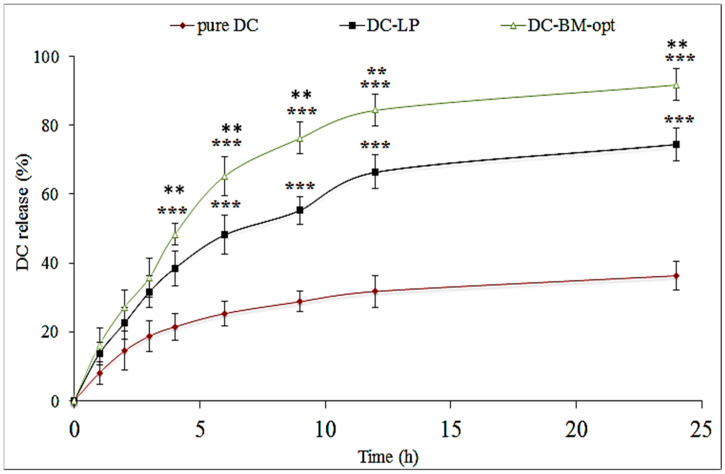
Release study of different treatment groups (pure diclofenac (DC), optimized diclofenac bilosomes (DC-BMs-opt), and diclofenac liposomes (DC-LP)). Study was performed in triplicate and data are shown as mean ± SD. Statistical analysis performed between each group and *p* < 0.05 considered significant. *** highly significant to pure DC; ** significant to pure DC-LP.

The release of DC-BMs-opt was analyzed and result was compared with DC-LP and pure DC. The data of release study are shown in Figure 5. DC-BMs-opt exhibited 91.82 ± 4.65% release in 24 h of study. The graph showed biphasic release behavior with an initial fast release and later sustained release. The fast release was due to presence of DC on surface of BM-opt and later the sustained release was found due to release of DC from DC-BMs matrix [16]. There was a significantly (*p* < 0.001) lower DC release achieved from DC-LP (74.54 ± 4.76%) and pure DC (36.32 ± 4.23). The liposomes (DC-LP) showed significantly (*p* < 0.001) higher DC release than the pure DC. The pure DC showed poor release due to poor solubility. The significant high release of DC was achieved from the BMs and LP due to enhanced DC solubility in presence of surfactant. There was also a significant difference in release achieved due to presence of bile salt in BMs. Bile salt showed a synergistic effect with used surfactant and can enhance greater solubility. 

The release profile of DC-BMs-opt was fitted into different kinetic models and data showed best fit model as the Korsmeyer–Peppas model (Table 4). The maximum regression value (R^2^ = 0.9354) confirms best fit. The exponent n-value was 0.58 (0.45 to 0.85) representing non-Fickian mechanism with dual release, i.e., diffusion and swelling release [40]. 

### 3.8. Ex Vivo Permeation Study

**Figure 6:**
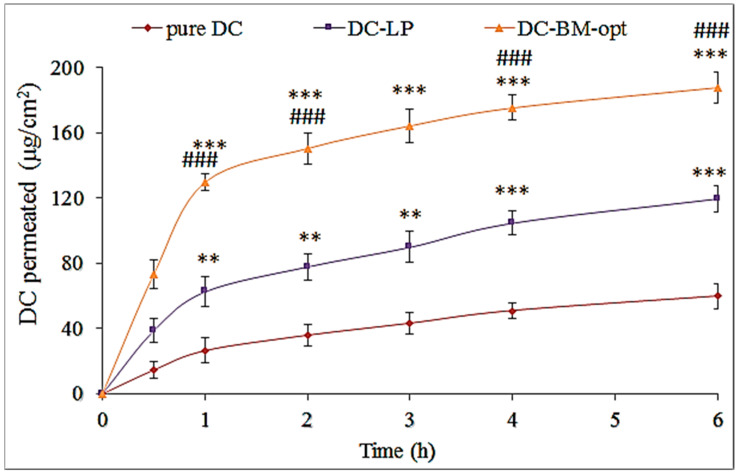
Permeation study of different treatment groups (pure diclofenac (DC), optimized diclofenac bilosomes (DC-BMs-opt), and diclofenac liposomes (DC-LP)). Study was performed in triplicate and result shown as mean ± SD. Statistical analysis performed between each group and *p* < 0.05 considered significant. *** highly significant to pure DC; ### significant to DC-LP; ** significant to pure DC.

The study of DC-BMs-opt was assessed to compare results with DC-LP and pure DC (Figure 6). The formulation DC-BMs-opt showed significantly (*p* < 0.001) higher permeation (187.59 ± 9.65 µg/cm^2^) than DC-LP (119.44 ± 10.06 µg/cm^2^) and DC-dispersion (59.52 ± 7.76 µg/cm^2^). It also exhibited significant (*p* < 0.05) 3.15-fold (31.26 µg/cm^2^/h) higher flux than pure DC (9.92 µg/cm^2^/h) and 1.57-fold higher than DC-LP (19.91 µg/cm^2^/h). DC-BMs-opt showed the APC of 2.08 × 10^−3^ cm/s, which was significantly higher (*p* < 0.05) than pure DC (6.6 × 10^−4^ cm/s) and DC-PL (1.33 × 10^−3^ cm/s). The pure DC showed lesser permeation due to the poor solubility and not being able to permeate across the biological membrane. The greater amount of DC permeates across the membrane from liposomes due to presence of lipid, cholesterol, and surfactant. The surfactant helps to solubilize drug and due to enhanced solubility, greater effective surface area is available for drug absorption. BMs were prepared with a special component as bile salt which helped to deform the vesicles and also helped to fluidize membrane, possibly because of interaction of phospholipid molecules with membrane layer [41]. Due to this property, it can permeate easily to smaller-sized membrane. The presence of cholesterol helps to extract lipid of membrane and act as a permeation enhancer. The larger amount of drug permeated from BMs. A size of more than 200 nm does not significantly affect permeation of drugs [42].

### 3.9. Bioavailability Study

**Figure 7:**
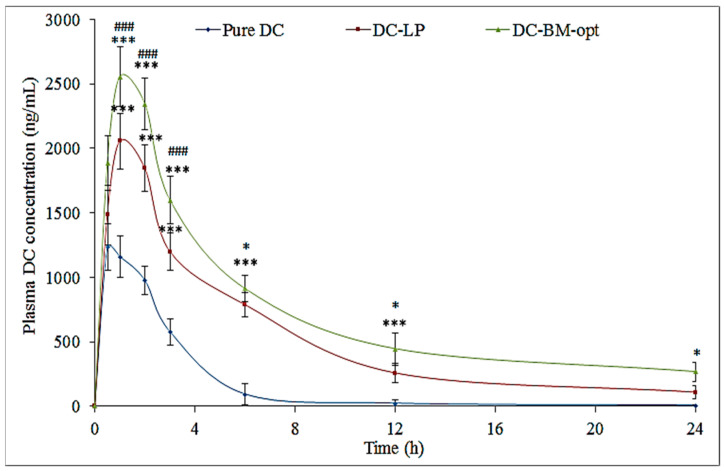
Bioavailability activity of the different treatment groups (pure diclofenac (DC), optimized diclofenac bilosomes (DC-BMs-opt), and diclofenac liposomes (DC-LP)). Study performed with six rats (n = 6) in each group and results shown as mean ± SD. Statistical analysis performed between each group and *p* < 0.05 considered significant. *** highly significant to pure DC; ### significant to DC- LP; * significant to pure DC and DC-LP.

The pharmacokinetic study of pure DC, DC-LP, and DC-BMs-opt was conducted and plasma concentration-time profile is expressed graphically in Figure 7. The result showed significant variation in each tested parameter. DC-BMs-opt showed a C_max_ value of 2654.76 ± ng/mL and was found to be 2.15-fold higher than pure DC (1232.34 ± ng/mL) and 1.29-fold higher than DC-LP (2054 ± ng/mL). The higher C_max_ was achieved due to nano-size of DC-BMs-opt, high permeability, and low first-pass metabolism. The difference was found to be highly significant (*p* < 0.001) compared to pure DC and DC-LP. DC-BMs-opt showed significant (*p* < 0.05) enhancement in AUC_0–t_ (22,340 ng. h/mL) and AUC_0–__∞_ (26,827.92 ng. h/mL) values. It was about 5.2 and 6.2-fold higher than pure DC (AUC_0–t_ of 4288.48 ng. h/mL and AUC_0–∞_ of 4319.12 ng. h/mL) and 1.43 and 1.56-fold higher than DC-LP (AUC_0–t_ of 15,564, AUC_0–∞_ of 17,170.09 ng. h/mL). The half-life (t_1/2_) of DC-LP and DC-BMs-opt was found to be higher (6.62 h and 8.39 h) than pure DC (1.95 h), which revealed that DC-BMs-opt was available for a longer time in circulation. DC-BMs-opt exhibited higher *T_max_* (1 h) than pure DC dispersion (30 min) due to an increase in solubility of DC in BM as well as LP. The elimination rate constant (Ke) for DC-BMs-opt was found to be significantly (*p* < 0.05) lower (0.08 h^−1^) than pure DC (0.22 h^−1^) and DC-LP (0.1 h^−1^) due to slow and prolonged drug release. The relative bioavailability of DC-BMs-opt showed 5.2-fold enhancement compared to pure DC and 1.43-fold higher compared to DC-LP. The higher bioavailability DC in DC-BMs-opt is due to increased DC solubility, longer circulation, lower first-pass metabolism, and higher uptake of BM by Peyer’s patch of M-cell of intestine [43].

### 3.10. Pharmacodynamic Study

**Figure 8:**
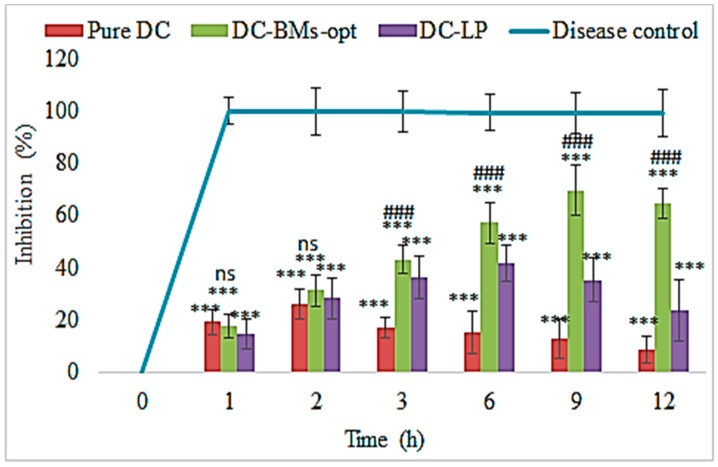
Anti-inflammatory activity of different treatment groups (pure diclofenac (DC), optimized diclofenac bilosomes (DC-BMs-opt), and diclofenac liposomes (DC-LP)) compared with disease control. Study performed with six rats (n = 6) in each group and result shown as mean ± SD. Statistical analysis performed between each group and *p* < 0.05 considered significant. *** highly significant to diabetic control; ### significant to pure DC and DC- LP; ns, non-significant to pure DC and DC-LP.

The anti-inflammatory activity of pure DC, DC-LP, and DC-BMs-opt was evaluated in carrageenan-induced model and results are expressed graphically in Figure 8. The disease control groups showed about 100% swelling. The pure DC, DC-LP, and DC-BMs-opt exhibited a significant effect in lowering paw edema. The pure DC, DC-LP, and DC-BMs-opt showed 26.23 ± 7.83%, 28.43 ± 5.67%, and 31.26 ± 6.13% reduction in paw edema after 2 h carrageenan injection, respectively. The pure DC-treated group showed maximum effect at 2 h, whereas DC-LP and DC-BMs-opt treated group showed a maximum effect up to 6 h and 9 h, respectively. There was a highly significant (*p* < 0.001) effect observed from DC-LP and DC-BMs-opt at 3 h, 6 h, 9 h, and 12 h in comparison to pure DC. At 12 h, maximum reduction was found to be 8.65 ± 3.87%, 23.76 ± 5.92%, and 64.76 ± 11.12% from pure DC, DC-LP, and DC-BMs-opt. At all-time points, a significant effect was observed from tested groups in comparison to disease control. DC-BMs-opt also exhibited a significant (*p* < 0.05) reduction in swelling than DC-LP. This high reduction in swelling was achieved due to high penetration capacity of DC-BMs-opt through intestinal mucosa. The nano-sized vesicle having a greater effective surface area, high circulation time, greater solubility and flexibility in presence of surfactant and bile salt led to greater absorption. Therefore, findings revealed that BMs may increase solubility and circulation of drugs which directly increases anti-inflammatory effect.

## 4. Conclusions

In the present study, DC-BMs were prepared by solvent evaporation method using sodium deoxycholate as bile salt. The formulations were optimized by Box–Behnken design to select optimum composition. The optimized formulation DC-BMs-opt showed a nano vesicle size and high encapsulation efficiency. The in vitro release and ex vivo permeation study showed a prolonged DC release with high permeation flux. The pharmacokinetic and pharmacodynamics study results revealed enhanced bioavailability and anti-inflammatory activity compared to pure DC and DC-LP. Further, prepared formulations need to be evaluated for clinical study. The findings of preclinical data need to be correlated with clinical data for better outcomes. We conclude from our findings that DC-BMs-opt is a promising oral drug delivery for treatment of inflammation.

## Figures and Tables

**Table 1:** Formulation composition and their effect on vesicle size and entrapment efficiency.

Code	Formulation Factor				Responses	
	CHO (A; %)	Lipid (B; %)	Surfactant (C; %)	Bile Salt (D; %)	Vesicle Size (nm; R_1_)	Entrapment Efficiency (%; R_2_)
1	0.1	0.5	0.5	1	169.34	50.23
2	0.5	0.5	0.5	1	284.53	88.23
3	0.1	1.5	0.5	1	275.34	75.43
4	0.5	1.5	0.5	1	380.14	92.32
5	0.3	1	0.3	0.5	328.12	65.02
6	0.3	1	0.7	0.5	260.34	84.12
7	0.3	1	0.3	1.5	291.65	76.32
8	0.3	1	0.7	1.5	218.54	81.32
9	0.1	1	0.5	0.5	243.45	58.43
10	0.5	1	0.5	0.5	335.27	85.12
11	0.1	1	0.5	1.5	203.16	72.02
12	0.5	1	0.5	1.5	297.65	94.03
13	0.3	0.5	0.3	1	248.24	57.23
14	0.3	1.5	0.3	1	357.00	75.12
15	0.3	0.5	0.7	1	200.43	77.32
16	0.3	1.5	0.7	1	298.14	93.11
17	0.1	1	0.3	1	256.76	60.23
18	0.5	1	0.3	1	345.65	81.87
19	0.1	1	0.7	1	194.54	70.21
20	0.5	1	0.7	1	290.00	90.24
21	0.3	0.5	0.5	0.5	242.87	57.25
22	0.5	1	0.7	1	341.54	83.51
23	0.3	0.5	0.5	1.5	210.00	77.87
24	0.3	1.5	0.5	1.5	306.23	85.23
25	0.3	1	0.5	1	270.21	79.01
26	0.3	1	0.5	1	270.43	79.09
27	0.3	1	0.5	1	269.36	80.87

**Table 2:** Statistical summary of best fit model for vesicle size (R_1_) and entrapment efficiency (R_2_).

Source	Vesicle Size (VS)	Entrapment Efficiency (EE)
Model	Linear	2F1
Adjusted R^2^	0.9900	0.9318
R^2^	0.9915	0.9580
Predicted R^2^	0.9867	0.8781
%CV	1.96	4.10
Adequate precision	85.98	21.95
SD	5.37	3.14

**Table 3:** ANOVA of best fitted designing model for vesicle size (Y_1_) and entrapment efficiency (Y_2_).

Vesicle Size (VS)					
Source	Sum of Squares	Mean Square	F-Value	*p*-Value Prob > F	Remark
Model (2nd order)	74,666.22	18,666.55	646.03	<0.0001	Significant
A-CHO	29,058.51	29,058.51	1005.69	<0.0001	--
B-Lipid	30,284.67	30,284.67	1048.13	<0.0001	--
C-Surfactant	11,128.26	11,128.26	385.14	<0.0001	--
D-Bile salt	4194.78	4194.784	145.17	<0.0001	--
Residual	635.66	28.89394	--	--	--
Lack of Fit	631.00	31.55001	13.52	0.0710	NS
Pure Error	4.66	2.333333	--	--	--
Total	75,301.89	--	--	--	--
**Entrapment efficiency (EE)**					
Model	3599.88	359.98	36.53	<0.0001	Significant
A-CHO	1779.01	1779.01	180.54	<0.0001	--
B-Lipid	778.43	778.43	79.00	<0.0001	--
C-Surfactant	544.05	544.05	55.21	<0.0001	--
D-Bile salt	239.41	239.41	24.29	0.0002	--
AB	110.25	110.25	11.18	0.0041	--
AC	0.66	0.66	0.06	0.7985	--
AD	6.25	6.25	0.63	0.4374	--
BC	1.22	1.22	0.12	0.7294	--
BD	91.58	91.58	9.29	0.0077	--
CD	49	49	4.97	0.0404	--
Residual	157.65	9.85	--	--	--
Lack of Fit	155.44	11.10	10.04	0.0942	NS
Pure Error	2.21	1.105	--	--	--
Total	3757.54	--	--	--	--

**Table 4:** Various kinetic release models and their regression value.

Type of Model	R^2^
Zero model	0.7344
First order	0.9257
Higuchi model	0.7744
Korsmeyer–Peppas	0.9354, n = 0.58
Hixon–Crowell model	0.8673

## Data Availability

Not Applicable.
